# Supplementary material for: Palmitoylated antigens for the induction of anti-tumor CD8+ T cells and enhanced tumor recognition
Source: Mol Ther Oncolytics. 2021 Apr 29;21:315–28. doi: 10.1016/j.omto.2021.04.009 (PMC8170356; doi:10.1016/j.omto.2021.04.009)
Supplement: Document S1. Figures S1–S3 [file mmc1.pdf]

OMTO, Volume *21*

Supplemental information

Palmitoylated antigens for the induction of anti-tumor CD8$^+$ T cells and enhanced tumor recognition

Dorian A. Stolk, Sophie K. Horrevorts, Sjoerd T.T. Schetters, Laura J.W. Kruijssen, Sanne Duinkerken, Eelco Keuning, Martino Ambrosini, Hakan Kalay, Rieneke van de Ven, Juan J. Garcia-Vallejo, Tanja D. de Gruijl, Sandra J. van Vliet, and Yvette van Kooyk

Supplementary Figure 1

A

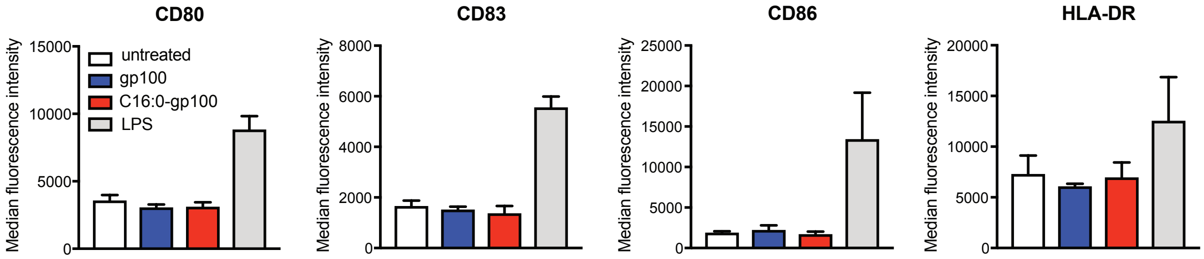

Figure S1. C16:0 peptide does not induce DC maturation (A) human moDC were incubated with unmodified peptide, C16:0 peptide, medium or LPS for 3hrs. Cells were washed and cultured o/n and stained for different maturation markers.

Supplementary Figure 2

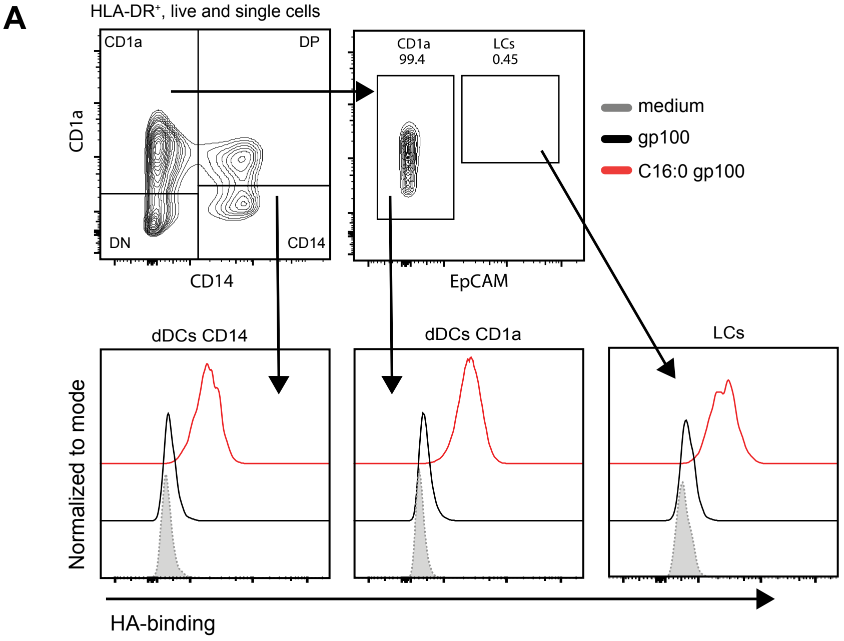

Figure S2. HA-tag detection in human skin crawl out dDCs. (A) Ex vivo human skin was intra-dermally injected with 30 µM unmodified gp100 or C16:0- p100 peptide in serum free medium. Punch biopsies were taken at the site of injection and cultured for 2 days. The crawl out cells were collected and stained for HLA-DR, CD1a, CD14, EpCAM and the HA-tag. Figure is representative for multiple individual experiments.

Supplementary Figure 3

A

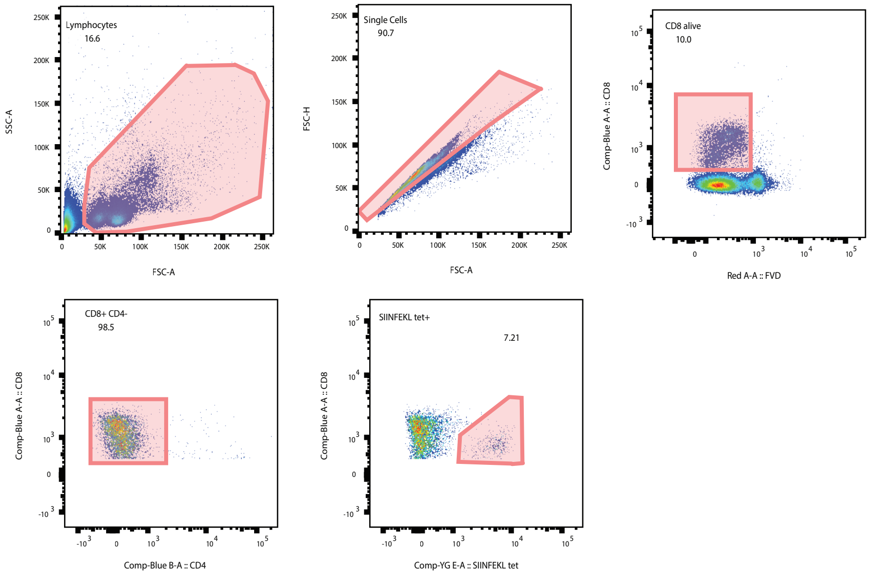

Figure S3. Gating strategy SIINFEKL+ CD8+ T-cells (A) Mouse blood or spleen was processed and stained with an antibody mxi containing CD8, CD4, Fixable Viability Dye (FVD), SIINFEKL tetramer.
