# Supplementary material for: Palmitoylated antigens for the induction of anti-tumor CD8+ T cells and enhanced tumor recognition
Source: Mol Ther Oncolytics. 2021 Apr 29;21:315–28. doi: 10.1016/j.omto.2021.04.009 (PMC8170356; doi:10.1016/j.omto.2021.04.009)
Supplement: Document S2. Article plus supplemental information [file mmc2.pdf]

Molecular Therapy
Oncolytics
Original Article

Palmitoylated antigens for the induction of anti-tumor CD8$^+$ T cells and enhanced tumor recognition

Dorian A. Stolk,[1,4] Sophie K. Horrevorts,[1,4] Sjoerd T.T. Schetters,[1,4] Laura J.W. Kruijssen,[1] Sanne Duinkerken,[1] Eelco Keuning,[1] Martino Ambrosini,[1] Hakan Kalay,[1] Rieneke van de Ven,[2,3] Juan J. Garcia-Vallejo,[1] Tanja D. de Gruijl,[2] Sandra J. van Vliet,[1] and Yvette van Kooyk[1]

[1]Amsterdam UMC, Department of Molecular Cell Biology and Immunology, Cancer Center Amsterdam, Amsterdam Institute for Infection & Immunity, Vrije Universiteit Amsterdam, 1081 HV Amsterdam, the Netherlands; [2]Amsterdam UMC, Department of Medical Oncology, Cancer Center Amsterdam, Amsterdam Institute for Infection & Immunity Vrije Universiteit Amsterdam, 1081 HV Amsterdam, the Netherlands; [3]Amsterdam UMC, Department of Otolaryngology/Head and Neck Surgery, Cancer Center Amsterdam, Vrije Universiteit Amsterdam, 1081 HV Amsterdam, the Netherlands

Induction of tumor-specific cytotoxic CD8$^+$ T cells (CTLs) via immunization relies on the presentation of tumor-associated peptides in major histocompatibility complex (MHC) class I molecules by dendritic cells (DCs). To achieve presentation of exogenous peptides into MHC class I, cytosolic processing and cross-presentation are required. Vaccination strategies aiming to induce tumor-specific CD8$^+$ T cells via this exogenous route therefore pose a challenge. In this study, we describe improved CD8$^+$ T cell induction and *in vivo* tumor suppression of mono-palmitic acid-modified (C16:0) antigenic peptides, which can be attributed to their unique processing route, efficient receptor-independent integration within lipid bilayers, and continuous intracellular accumulation and presentation through MHC class I. We propose that this membrane-integrating feature of palmitoylated peptides can be exploited as a tool for quick and efficient antigen enrichment and MHC class I loading. Importantly, both DCs and non-professional antigen-presenting cells (APCs), similar to tumor cells, facilitate anti-tumor immunity by efficient CTL priming via DCs and effective recognition of tumors through enhanced presentation of antigens.

INTRODUCTION

Despite the success of immune checkpoint inhibitor (ICI) therapy, numerous advanced-stage melanoma patients still do not experience clinical benefit.[1–3] Cancer vaccines could potentially initiate *de novo* or boost existing anti-tumor T cell responses in these patients. However, therapeutic vaccination to treat cancer has proved to be challenging, as the induction of adequate immune responses needs to overcome the immune dampening mechanisms that sustain the disease. One of the hurdles in vaccine-induced anti-tumor immunity is the induction of robust cytotoxic CD8$^+$ T lymphocyte (CTL) responses, which is vital for tumor killing. Dendritic cells (DCs) are key players in this process, as they have the unique capacity to take up exogenous antigens for presentation to both CD4$^+$ T helper (Th) cells and CTLs in the lymph nodes.[4] The intracellular mechanisms of exogenously derived antigen cross-presentation on major histocompatibility complex (MHC) class I by DCs, and the identification of the main cross-presenting DC subset in humans and mice, have been major topics of research for many years. Murine *in vivo* studies revealed that cross-presentation is mostly executed by *Batf3*-dependent conventional DCs (cDC1s).[5] In contrast, human *in vitro* studies have shown that beside cDC1s, also cDC2s, plasmacytoid DCs (pDCs), dermal DCs (e.g., CD1a$^+$ dDCs and CD14$^+$ dDCs), human *in vitro*-generated monocyte-derived DCs (moDCs), and Langerhans cells (LCs) (e.g., CD1a$^+$Langerin$^+$ EpCAM$^+$) are able to cross-present exogenous antigen.[6–11] Efficient antigen delivery to DCs is important to reach optimal induction of T cell responses, and different (receptor) targeting strategies or peptide modifications have already been investigated. DC-specific receptors, for example the C-type lectin receptors (CLRs) DEC205, CLEC9A, DC-SIGN, and Langerin, have been extensively studied as targets for vaccine delivery.[9,12–14] Multiple CLRs were shown to be promising targets by facilitating the induction of efficient cross-presentation of tumor antigens; however, these targeting strategies were mostly limited to selected DC subsets.[15] Another potential drawback of using receptors as targeting molecules is related to inherent receptor dynamics. Non-recycling CLRs, such as DC-SIGN, can only take up a limited amount of antigen.[16] Moreover, not all DC targeting ligands are routed into efficient cross-presentation pathways.[17]

Interestingly, already in the 1980s, Hopp and colleagues[18] found that conjugation of a fatty acid moiety to a hepatitis virus epitope

Received 5 April 2021; accepted 24 April 2021;
https://doi.org/10.1016/j.omto.2021.04.009.

[4]These authors contributed equally

Correspondence: Yvette van Kooyk, Amsterdam UMC, Department of Molecular Cell Biology and Immunology, Cancer Center Amsterdam, Amsterdam Institute for Infection & Immunity, Vrije Universiteit Amsterdam, 1081 HV Amsterdam, the Netherlands.
E-mail: y.vankooyk@amsterdamumc.nl

This is an open access article under the CC BY license (http://creativecommons.org/licenses/by/4.0/).

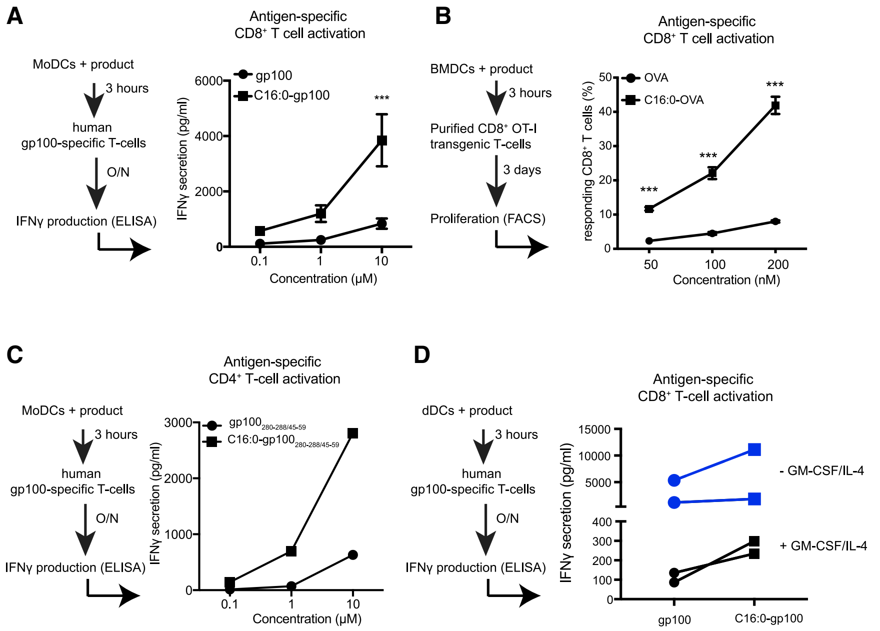

Figure 1. Palmitic acid-conjugated peptides induce enhanced T cell activation *in vitro* and *in vivo*

(A) moDCs were pulsed with different concentrations of gp100$_{280–288/45–59}$ or C16:0 gp100$_{280–288/45–59}$ for 3 h and co-cultured o/n with gp100-specific CD8^{+} T cells. IFNɣ secretion was used as a measure for T cell activation. Data are presented as mean ± SEM of six individual experiments, measured in triplicate. Statistical analyses were performed using a two-way ANOVA with a Sidak post hoc multiple comparison test. ***$p < 0.001$. (B) OVA$_{257–264/323–339}$- and C16:0 OVA$_{257–264/323–339}$-loaded BMDCs were co-cultured with OT-I T cells for 3 days. Mean ± SEM measured in triplicate. Data are representative of three individual experiments. Statistical analyses were performed using a two-way ANOVA with a Sidak post hoc multiple comparison test. **$p < 0.01$, ***$p < 0.001$. (C) moDCs were pulsed with different concentrations of gp100$_{280–288/45–59}$ or C16:0 gp100$_{280–288/45–59}$ for 3 h and co-cultured o/n with gp100-specific CD4^{+} T cells. IFNɣ secretion was used as a measure for T cell activation. Data are presented as mean ± SEM measured in triplicate and representative of three individual experiments (D) gp100$_{280–288/45–59}$ and C16:0 gp100$_{280–288/45–59}$ (30 μM) were injected intradermally into human skin with (black line) or without (blue line) GM-CSF/IL-4. At the site of injection punch biopsies were taken and cultured for 2 days. The migrated skin APCs were collected and co-cultured o/n with gp100-specific CD8^{+} T cells. IFNɣ secretion was used as a measure for T cell activation. Data of two individual donors per condition are shown.

significantly improved antibody (Ab) responses. Since then, many studies have been conducted studying peptides modified with a single palmitic acid (palmitoylation) and highlighted their potential for increasing immunogenicity of peptide-based vaccination strategies.[19–22] However, the underlying mechanisms for enhanced immunogenicity are not entirely elucidated, and the characteristics of the position and spacer sequence of the lipid vector seem to determine its processibility.[23] As such, peptide mono-palmitoylation on lysine residues on either the C- or N-terminus of two different synthetic lipopeptides was shown to induce efficient CD8^{+} T cell activation, but interestingly they were processed via different routes.[24] Moreover, direct mono-palmitoylation to the N-terminal backbone of a viral peptide was unable to induce CTLs.[25] Additional detailed studies are warranted to further elucidate the immunogenicity of mono-palmitoylated antigens and the associated processing pathways to further optimize vaccination strategies.

In this study, we describe that mono-palmitoylation directly on the N-terminus of synthetic long peptide enhances CD8^{+} and CD4^{+} T cell activation and induces *in vivo* tumor suppression. We show that the superior cross-presentation of palmitoylated peptides results from efficient cytosolic proteasome-dependent degradation, but also vacuolar processing. Additionally, we demonstrate that mono-palmitoylated peptides effectively integrate in membranes and are efficiently internalized by DCs independent of recycling receptors. This membrane integration and cross-presentation capacity is not exclusively attributed to DCs, but also to non-professional antigen-presenting cells (APCs), such as tumor cells or tumor-derived vesicles. Moreover, loading of tumor cells with palmitoylated peptide enhances sensitivity to CTL-mediated killing. We therefore propose a novel application of the use of palmitoylated peptides, in which they can be exploited as a tool for enrichment of peptide/MHC class I complexes and subsequent activation of CTLs, following effective recognition and killing of tumor cells.

RESULTS

Palmitoylated peptides are efficiently presented by mouse and human APC subsets *in vitro*, resulting in potent CD8^{+} and CD4^{+} T cell activation

To assess the anti-tumor potential of modified antigens, synthetic long peptides containing the dominant MHC class I and II epitopes of the human melanoma antigen gp100 (gp100$_{280–288/45–59}$) and the mouse model antigen ovalbumin (OVA$_{257–264/323–339}$) were synthesized and conjugated to palmitic anhydride (C16:0), creating mono-palmitoylated peptides. Co-culture of mono-palmitoylated C16:0 peptide-pulsed human moDCs (Figure 1A) or mouse bone marrow-derived DCs (BMDCs) (Figure 1B) with either gp100-specific or OVA-specific (OTI) CD8^{+} T cells, respectively, showed a highly robust induction of CD8^{+} T cells, as reflected by secretion of interferon (IFN)γ and T cell proliferation, that was significantly increased compared to peptide pulsed conditions. Similar results were observed with CD4^{+} gp100-specific T cells, where palmitoylated peptide outperformed unmodified peptide in the induction of antigen-specific T cells (Figure 1C). It has previously been suggested that palmitoylated antigens induced

Toll-like receptor (TLR)-mediated DC activation.[26] Analysis on the activation status of DCs revealed that incubation of moDCs with palmitoylated peptide did not induce increased expression on the maturation markers CD80, CD83, CD86, and human leukocyte antigen (HLA)-DR (Figure S1). Therefore, increased T cell activation was not related to phenotypic changes in DCs. Next, the potency of C16:0 peptides in terms of delivering antigens to human skin-resident APCs as a vaccination strategy was evaluated. To this end, gp100 C16:0 and unmodified gp100 peptides were injected into the dermis of human *ex vivo* skin explants with or without supplementation of the often used adjuvant combination granulocyte-macrophage colony-stimulating factor (GM-CSF)/interleukin (IL)-4.[27] Next crawl-out cells, which have a mixed phenotype,[28] were harvested 48 h after injection, stained for the presence of C16:0 peptide, or co-cultured with gp100-specific $CD8^+$ T cells. Interestingly, in the context of the human skin tissue microenvironment, vaccination with C16:0 gp100 peptide led to uptake in multiple skin DC subsets (Figure S2) and resulted in improved presentation to gp100-specific $CD8^+$ T cells compared to unmodified gp100 peptides, as reflected by increased levels of IFNγ in the supernatant (Figure 1D). Collectively, these data show that multiple DC subsets efficiently process and present C16:0-modified peptides, resulting in the activation and induction of antigen-specific $CD8^+$ and $CD4^+$ T cells *in vitro* and *ex vivo*.

C16:0 peptides induce robust $CD8^+$ T cell responses *in vivo* and enhanced tumor suppression

After validating in multiple *in vitro* assays that C16:0 peptides were superior compared to unmodified peptides in facilitating efficient MHC class I and MHC class II loading and subsequent presentation to T cells, we aimed to elucidate the *in vivo* effects of C16:0 vaccination. C57BL/6 mice were immunized subcutaneously (s.c.) with C16:0 or unmodified OVA peptide supplemented with adjuvant (monoclonal anti-CD40). At 7 days post-immunization (p.i.), blood-derived cells were analyzed by fluorescence-activated cell sorting (FACS) for antigen-specific $CD8^+$ T cells using H-$2k^b$-phycoerythrin (PE) SIINFEKL tetramer (tet) (see gating strategy in Figure S3). As shown by the frequency of $SIINFEKL^+$ $CD8^+$ T cells, immunization with C16:0 peptides led to a significantly higher induction of antigen-specific T cells compared to peptide-immunized mice (Figure 2A, left panel). This difference was still present 21 days after an additional boost that was given 14 days after the initial immunization (Figure 2A, right panel). As a final test to assess whether cross-presentation of palmitoylated synthetic long peptides to $CD8^+$ T cells would lead to increased tumor cell killing *in vivo*, B16-OVA tumor cells were injected s.c. into immunocompetent C57BL/6 mice. Mice were divided into groups based on equal palpable tumor size 9 days after tumor inoculation (Figure 2B) and vaccinated with either palmitoylated or unmodified constructs supplemented with adjuvant (agonistic CD40 Ab). An additional boost vaccination was given 14 days after the initial vaccination. Remarkably, there was an apparent reduction in tumor growth in mice immunized with C16:0 peptides compared to peptide counterparts (Figure 2C), although no differences were found in the frequency of antigen-specific $CD8^+$ T cells in the blood on day 7 after vaccination (data not shown). On average, tumors of C16:0-vaccinated mice grew 9.68 mm^3 per day compared to 17.27 mm^3 in peptide-immunized mice as analyzed with the linear regression model (Figure 2D). Also, survival of mice vaccinated with palmitoylated antigens increased (Figure 2E), since four of six mice were still alive in the C16:0-immunized group 49 days after the start of treatment compared to one of six mice from the unmodified peptide group (Figure 2F). Overall, these data confirm a beneficial effect of mono-palmitoylation of peptides on anti-tumor responses *in vivo*.

Palmitoylated peptides follow a different processing route than unmodified peptides and can be cross-presented by *Batf3* knockout (KO) DCs

Since increased T cell activation was not related to phenotypic changes in DCs (Figure S1), we further looked into possible underlying mechanisms leading to enhanced cross-presentation by investigating the processing pathway of C16:0 peptides. Hereto, moDCs were treated with different inhibitors interfering with peptide processing before loading with C16:0 or unmodified peptides. After loading, moDCs were co-cultured with gp100-specific $CD8^+$ T cells, and IFNγ levels were measured as a readout for cross-presentation. Chloroquine was used as an inhibitor for endosomal acidification, the leupeptin and cathepsin S inhibitor for blocking of proteolysis, bortezomib to interfere with the proteasome, and primaquine was used to hamper surface-directed transport of recycling endosomes (Figure 3A). Using this variety of inhibitors allowed us to determine whether the processing of internalized exogenous C16:0 peptides differed from the processing of unmodified peptides. Inhibition of the proteasome with bortezomib exerted a significant inhibitory effect on processing and presentation of C16:0 peptides to T cells compared to unmodified peptide (Figure 3B). Moreover, blocking of proteolysis by the cathepsin S inhibitor (Figure 3C) and blocking of endosomal acidification via chloroquine (Figure 3D) affected presentation of C16:0 peptides, while no differences were found for the presentation of unmodified peptide. In contrast, reducing surface-directed transport of recycling endosomes by primaquine only affected the presentation of unmodified peptides (Figure 3E). No differences in IFNγ secretion were found after co-culture of moDCs and T cells pre-incubated with leupeptin (data not shown). Since trimmed peptides need to be loaded onto MHC class I molecules and this can either occur in the endosomes or the endoplasmic reticulum (ER),[29] we used brefeldin A (BFA) to block transport from the ER to the Golgi,[30] thereby inhibiting the ER-dependent MHC class I loading implicated in the cytosolic pathway. Surprisingly, we did not observe inhibition of cross-presentation by the moDCs pulsed with the C16:0 peptide, whereas cross-presentation of the unmodified peptide was inhibited by BFA (Figure 3F). Taken together, these data suggest that the superior antigen presentation of C16:0 peptides is the result of efficient cytosolic and vacuolar processing that is different from unmodified peptide. Unmodified peptides seem to form a peptide/MHC class I complex in vacuoles, illustrated by the reduction in cross-presentation when surface transport of recycling endosomes was blocked with primaquine, but interestingly unmodified peptides also seem to be loaded onto MHC class I in the ER after processing via the cytosolic pathway as seen by the BFA-induced inhibition of cross-presentation.

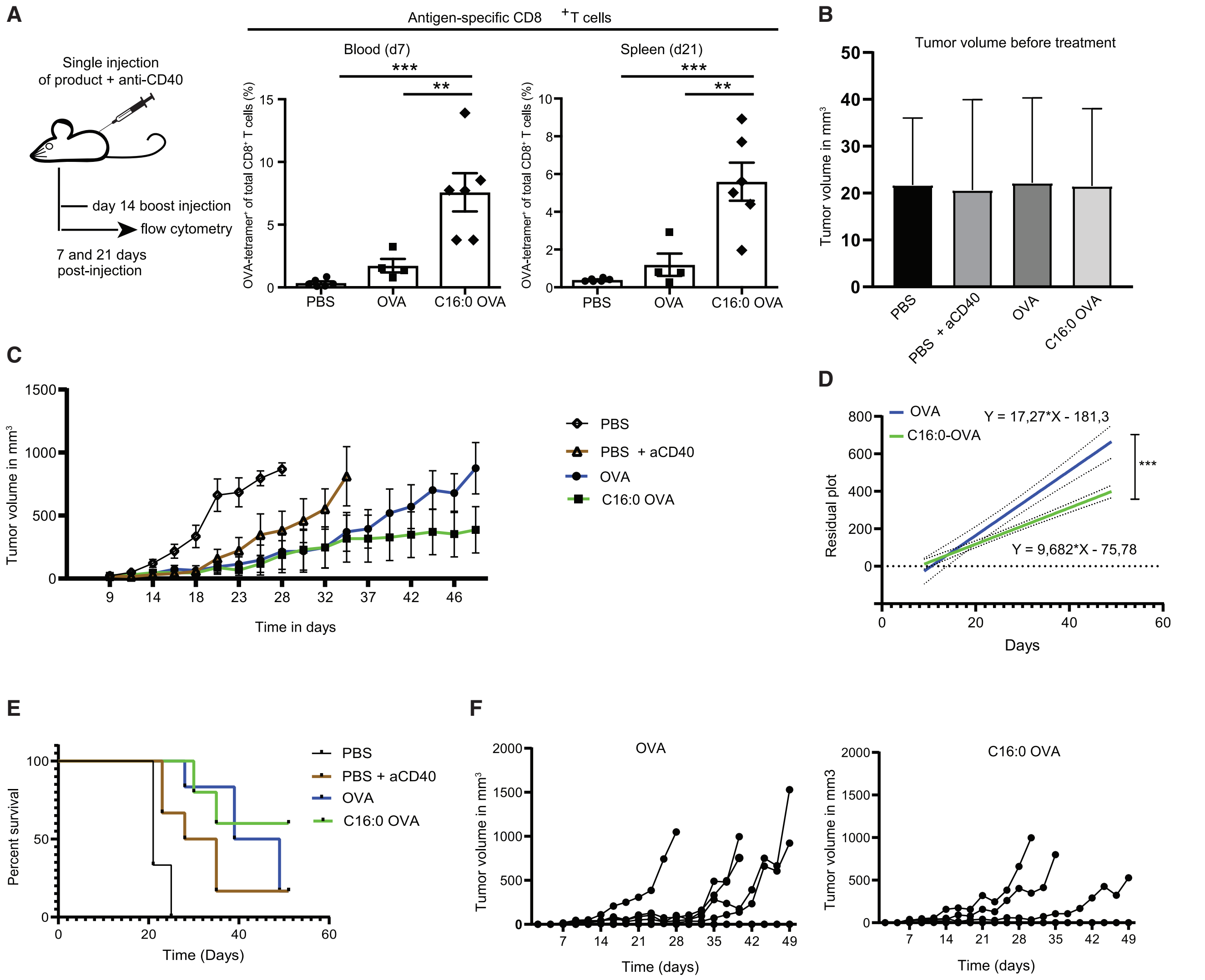

Figure 2. C16:0 peptides induce robust CD8+ T cell responses *in vivo* and enhance tumor suppression

(A) C57BL/6 mice were vaccinated with OVA$_{257–264/323–339}$ or C16:0 OVA$_{257–264/323–339}$ in combination with anti-CD40. At day 7 (blood) and day 21 (spleen) antigen-specific CD8^{+} T cells were detected by tetramer (tet) staining and cells were gated on live/single cells/CD4^{-}/CD8^{+}/tet^{+}. Data points represent indivual mice ± SEM. Statistical analyses were performed using a one-way ANOVA with a Tukey post hoc multiple comparison test. **$p < 0.01$, ***$p < 0.001$. (B) Tumor volume per group at start of s.c. vaccination. Data represent mean ± SD of six mice per group. (C) Tumor volume over time in the different treatment groups as measured by caliper. Data are shown as mean ± SEM of six individual mice. (D) Linear regression analysis of tumor growth of OVA$_{257–264/323–339}$- and C16:0 OVA$_{257–264/323–339}$-immunized mice. ***$p < 0.001$. (E) Survival plot of mice per group. (F) Individual tumor growth curves of OVA$_{257–264/323–339}$ (left panel) and C16:0 OVA$_{257–264/323–339}$ (right panel)-immunized mice. In the OVA$_{257–264/323–339}$ group, five of six mice developed a tumor >800 mm^{3}, while two of six mice developed a tumor >800 mm^{3} in the C16:0 OVA$_{257–264/323–339}$ group.

If C16:0 peptides use a cross-presentation route that is different from unmodified peptides, we reasoned that cross-presentation of mono-palmitoylated peptides might not be reliant on the *Batf3*-dependent professional cross-presenting DCs.[5] To test this hypothesis, CD11c^{+} DCs from spleens of wild-type (WT) and *Batf3* KO mice were isolated, loaded with C16:0 or unmodified OVA peptides, and co-cultured with OVA-specific CD8^{+} T cells (OT-I) (Figure 3G). Interestingly, C16:0 peptide was efficiently cross-presented by CD11c^{+} DCs from *Batf3* KO mice, whereas presentation of unmodified peptides was significantly reduced, as reflected by CD8^{+} T cell activation (Figure 3H). Overall, we concluded that the preferential processing route of C16:0 and unmodified peptides is not shared and that C16:0 can be efficiently presented by APCs from *Batf3* KO mice.

Palmitic acid-conjugated peptides efficiently integrate in membranes of DCs and are quickly internalized independent of recycling receptors

After assessing efficient cross-presentation of C16:0 and finding that C16:0 peptides have a unique processing pathway compared to unmodified peptide, we next looked into the initial step, that is, cell entry

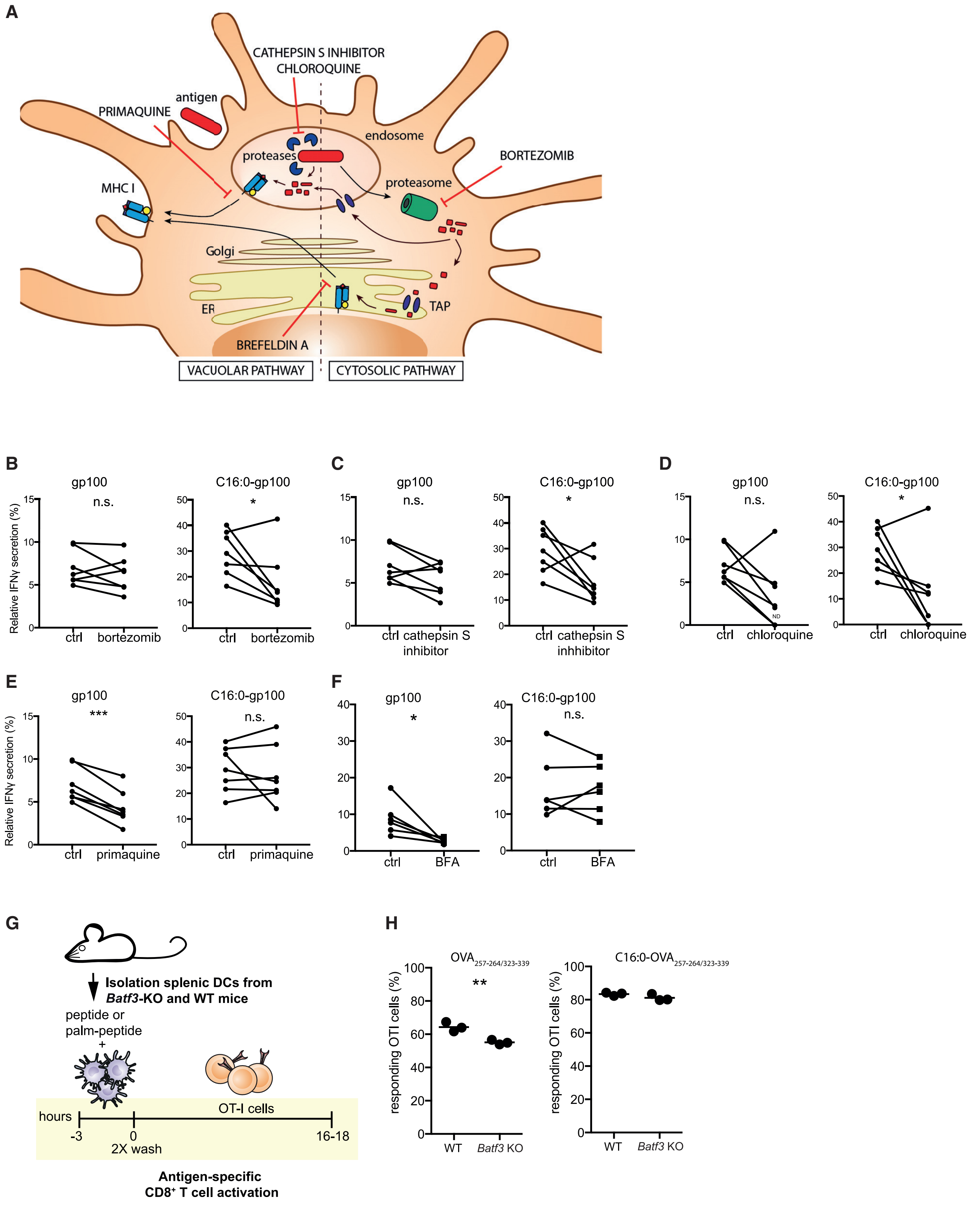

(legend on next page)

and internalization. To allow visualization of C16:0 peptides, we generated gp100 peptides that include the influenza-derived hemagglutinin (HA) peptide sequence, to which high-affinity Abs are available. HA has an advantage over fluorochrome conjugation by not altering tertiary structure through modification of amino acid side chains. Incubation of moDCs with C16:0 peptides at 37°C revealed that the HA signal of C16:0 peptides was preserved over time, showing no decay of signal, suggesting that association of C16:0 peptides with DCs was a continuous process (Figure 4A). Moreover, this signal was superior compared to the control peptide. However, until this point the exact localization of the C16:0 peptide, i.e., membrane or intracellular, was unclear. Therefore DCs were incubated for 45 min at 4°C and stained with anti-HA Ab conjugated to Alexa Fluor (AF) 647 for detection of peptide membrane binding and with anti-HA AF488 for intracellular detection of peptide. To check whether staining with anti-HA-AF647 occupied all the membrane binding sites of C16:0 peptide, DCs were fixed after staining and incubated with anti-HA-AF488. No staining with anti-HA-AF488 was detected, confirming saturation of anti-HA-AF647 in the first staining step (Figure 4B, left panel). Subsequently, DCs were permeabilized to allow staining of C16:0 peptides located in the intracellular space with anti-HA-AF488. A strong signal for C16:0 peptide was measured on the cell surface by anti-HA-AF647 and intracellularly by anti-HA-AF488 (Figure 4B, middle and right panels), indicating efficient membrane integration, but also internalization already at 4°C. Using stimulated emission depletion (STED) super-resolution confocal microscopy, we indeed confirmed strong and uniform integration of C16:0 peptide in the cell membrane of DCs at 4°C (Figure 4C), suggesting direct association of the lipid tail of the peptide with the lipid bilayer of the cell. Unmodified peptide was not detectable in the membrane of DCs. To investigate whether the continuous uptake and internalization of the C16:0 peptide could be explained by cellular receptors known for quick recycling, such as the mannose receptor (MR), moDCs were depleted for glucose by 2-deoxy-D-glucose (2DG) 30 min prior to the pulse with C16:0 peptide. Unconjugated anti-MR Ab was used as a control. After incubating for 45 min at 4°C to only allow binding, cells were fixed and stained for the MR or HA tag (Figure 4D). Reduction of surface expression levels of MR after 2DG pre-treatment suggested efficient depletion of glucose and reduced receptor recycling.[31] However, no reduction in binding of C16:0 peptide was observed after glucose depletion, implying that the continuous uptake of C16:0 peptide was not dependent on the binding to a fast-recycling receptor. Additionally, incubation of DCs at 37°C after a loading step with C16:0 peptide at 4°C and washing of the product revealed that the total amount of detectable HA tag decreased over time, not only implying rapid internalization, but also rapid processing of the C16:0 peptide (Figure 4E). Taken together, these data suggest that next to a differential processing, the increased binding and uptake of C16:0 peptides compared to unmodified peptide facilitates increased cross-presentation and anti-tumor responses. Moreover, it seems that internalization of C16:0 peptides is not mediated via recycling receptors or active rearrangement of membranes, but that it is rather an energy-independent, immediate, and continuous process.

Palmitoylated peptides as a tool for antigen enrichment in tumor cells and tumor-derived apoptotic vesicles for enhanced presentation on MHC class I and subsequent recognition by CTLs

The fact that palmitoylated peptides possess the capability to integrate into membranes of DCs in an energy and recycling receptor-independent manner led us to hypothesize that synthetically palmitoylated peptides possess a membrane binding ability that is applicable to any lipid bilayer. Therefore, next to murine and human DCs, the human melanoma cancer cell lines Mel-JuSo and Mel-BRO, the leukemic THP-1 cell line, and the B cell lymphoblastoid cell line JY were incubated with C16:0 or unmodified peptide for 45 min at 4°C. While the unmodified peptide was not detectable, a strong anti-HA signal was detected in all cells pulsed with C16:0 peptides (Figure 5A), confirming our hypothesis that palmitoylated peptides possess a unique capability to integrate into lipid bilayers. Next, we postulated that, because of this bilayer integrating feature and their efficient loading into MHC class I, C16:0 peptides might be used as an efficient tool to enrich tumor cells with MHC class I/peptide complexes. Since the effector phase of T cell-mediated anti-tumor responses relies on the presentation of tumor antigens in the context of MHC class I by the tumor cells, and insufficient presentation of antigenic peptides in MHC class I hampers CTL-mediated tumor killing, loading of tumor cells with C16:0 peptides would perhaps allow for improved tumor recognition and killing by antigen-specific $CD8^+$ T cells. Indeed, loading of human leukemic THP-1 cells (Figure 5B), as well as murine glioblastoma GL261 cells (Figure 5C), with C16:0 peptide to enrich gp100 epitope expression on MHC class I increased killing of tumor cells by gp100-specific MHC class I-restricted $CD8^+$ T cells. Finally, we reasoned that antigen enrichment could also be established using non-cellular lipid membranes. Especially tumor-derived extracellular vesicles provide a possible attractive source to induce patient-specific anti-tumor-immunity, whose antigenic content could be further enhanced by C16:0 peptides. To test this hypothesis, apoptotic cell-derived extracellular vesicles

Figure 3. The addition of a single palmitic acid affects the processing pathway of antigens

moDCs were treated with different inhibitors or DMSO control (ctrl) 30 min prior to and during the 3 h antigen pulse. Thereafter, moDCs were washed and co-cultured o/n with gp100-specific $CD8^+$ T cells. IFNγ secretion was determined by ELISA as a measure of T cell activation. (A) Illustration of interference in antigen processing by the different inhibitors. (B–F) Relative IFNγ secretion induced by co-culture of gp100-specific $CD8^+$ T cells with $gp100_{280–288/45–59}$- or C16:0 $gp100_{280–288/45–59}$-pulsed moDCs that were pre-treated with (B) bortezomib, (C) cathepsin S inhibitor, (D) chloroquine, (E) primaquine, and (F) brefeldin A. Data are represented as relative to the short peptide control after subtraction of background (IFNγ levels of unpulsed moDCs). Symbols represent the mean of triplicates of seven donors. Statistical analyses were performed with a paired Student's t test. *p < 0.05, ***p < 0.001. (G) Overview of experimental setup for antigen presentation with DCs from *Batf3* KO mice. (H) Proliferation of OT-I cells after co-culture with C16:0 or unmodified peptide in WT and *Batf3* KO BMDCs. Statistical analysis was performed with a Student's t test. **p < 0.01.

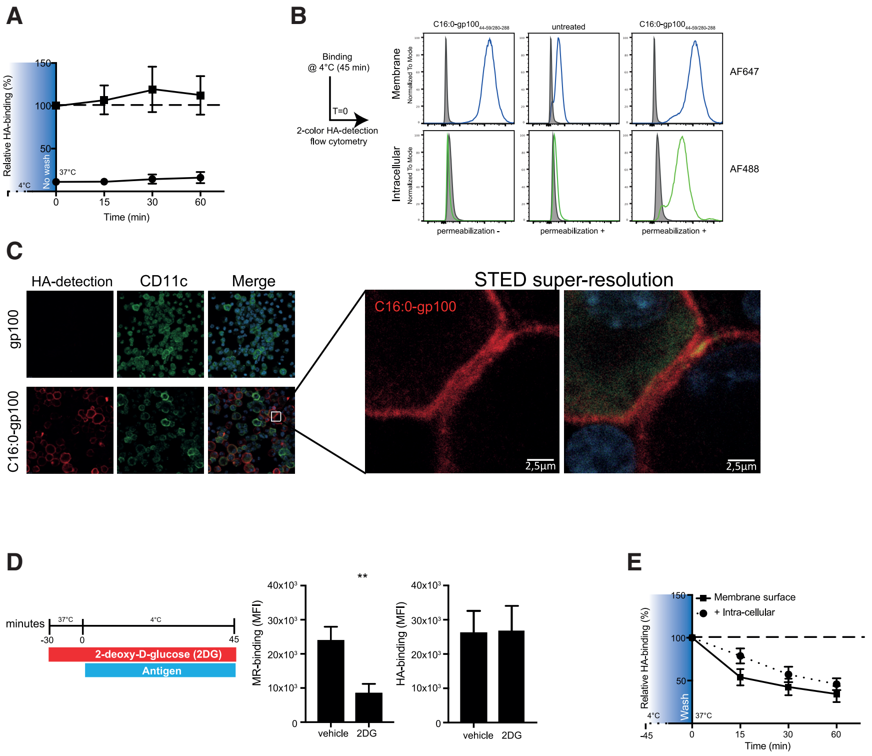

Figure 4. Palmitoylation of peptides is a membrane-targeting moiety that allows for continuous loading and processing

(A) HA signal at different time points in fixed and permeabilized moDCs that were pulsed with 10 μM gp100$_{280–288/45–59}$ (black circles) or C16:0 gp100$_{280–288/45–59}$ (black squares) for 45 min at 4°C, after which moDCs were transferred to a 37°C incubator without washing away the product. Data are shown as mean ± SEM of three individual experiments. (B) Membrane-bound HA signal (AF647, upper panel) and intracellular HA signal (AF488, lower panel) of moDCs that were pulsed with C16:0 peptide or left untreated for 45 min at 4°C with and without permeabilization. (C) Visualization of the membrane localization of the C16:0 gp100$_{280–288/45–59}$-HA peptide in BMDCs after a 45-min incubation at 4°C determined by STED confocal microscopy. (D) Mannose receptor (MR) and C16:0 peptide HA detection in moDCs treated with glucose depletion medium prior to the antigen pulse (45 min at 4°C). Data are shown as mean ± SEM of four individual experiments. Statistical analyses were performed with a paired Student's t test. $^{**}p < 0.01$. (E) Decay of membrane and total (membrane + intracellular) HA signal at different time points in moDCs that were pulsed with 10 μM C16:0 gp100$_{280–288/45–59}$. Graphs are presented as mean ± SEM of three individual experiments.

(ApoEVs) were generated from a gp100-expressing melanoma tumor cell line (Mel-JuSo)[32] and pulsed with C16:0 peptide for 30 min, before their addition to moDCs (Figure 5D). moDCs loaded with C16:0 peptide-enriched ApoEVs induced superior tumor antigen-specific T cell activation (Figure 5E). These data therefore confirm that the membrane-integrating capacity of C16:0 is extended into enhanced cross-presentation, even by non-professional APCs, such as tumor cells, and additionally these results provide evidence that C16:0 peptides can be used to enrich tumor-derived vesicles with antigens. C16:0 peptides thus provide an attractive and efficient platform to increase MHC class I peptide presentation, recognition, and subsequent killing by antigen-specific T cells.

DISCUSSION

Presentation of antigen-derived peptides in MHC class I molecules is vital for the induction and priming of CTL-mediated anti-tumor immunity and for the subsequent recognition and killing of the tumor cells by CTLs. For the generation of strong effector CTLs the efficiency of DCs to cross-present antigen is crucial, and this has been a hurdle in many vaccination studies. Moreover, proper and sufficient presentation of antigens on tumor cells is a second deficit, limiting CTL recognition and killing activity. In this study, we show that the conjugation of a single palmitic acid (C16:0) to a synthetic long antigenic peptide greatly improved the binding and uptake, which was followed by a different intracellular processing route for loading in MHC class I, leading to strong CTL activation and tumor suppression. Since the palmitoylation of antigen allows non-targeted association with the cell membrane, the increased antigen presentation is achieved in multiple DC subsets. Moreover, C16:0 peptide loading allowed for an increased peptide/MHC complex density on the surface of tumor cells, resulting in better MHC class I-mediated recognition by antigen-specific T cells, in turn leading to improved antigen-specific tumor killing. Thus, modification of antigen-encoding peptides with C16:0 provides a multi-pronged improvement of antigen-specific CTL responses, CD4^{+} T cell activity, and recognition of tumor cells, affecting both the induction and effector phase of anti-cancer immunity.

The increased immunity of palmitoylated peptides has been described before, and interestingly some palmitoylated vaccine formulations have also shown promising result in clinical trials.[33–37] However, palmitoylation exists in different forms, and most previous studies looked at di-or tri-palmitoylation or mono-palmitoylation that was established using a lysine-palmitic acid building block or direct linkage at the C-terminus.[19–25] In the present study, we investigated direct mono-palmitoylation to the amino acid at the N-terminal end, which is a more cost-effective method, and we found that multiple DC subsets could efficiently present mono-palmitoylated antigens, resulting in increased induction of antigen-specific CD8^{+} and CD4^{+} T cell responses. Additionally, we demonstrate that immunization with palmitoylated peptides enhanced suppression of B16-OVA tumors *in vivo*.

Mono-palmitoylated peptide vaccines were initially developed to create a self-adjuvanting peptide vaccine, through the supposed

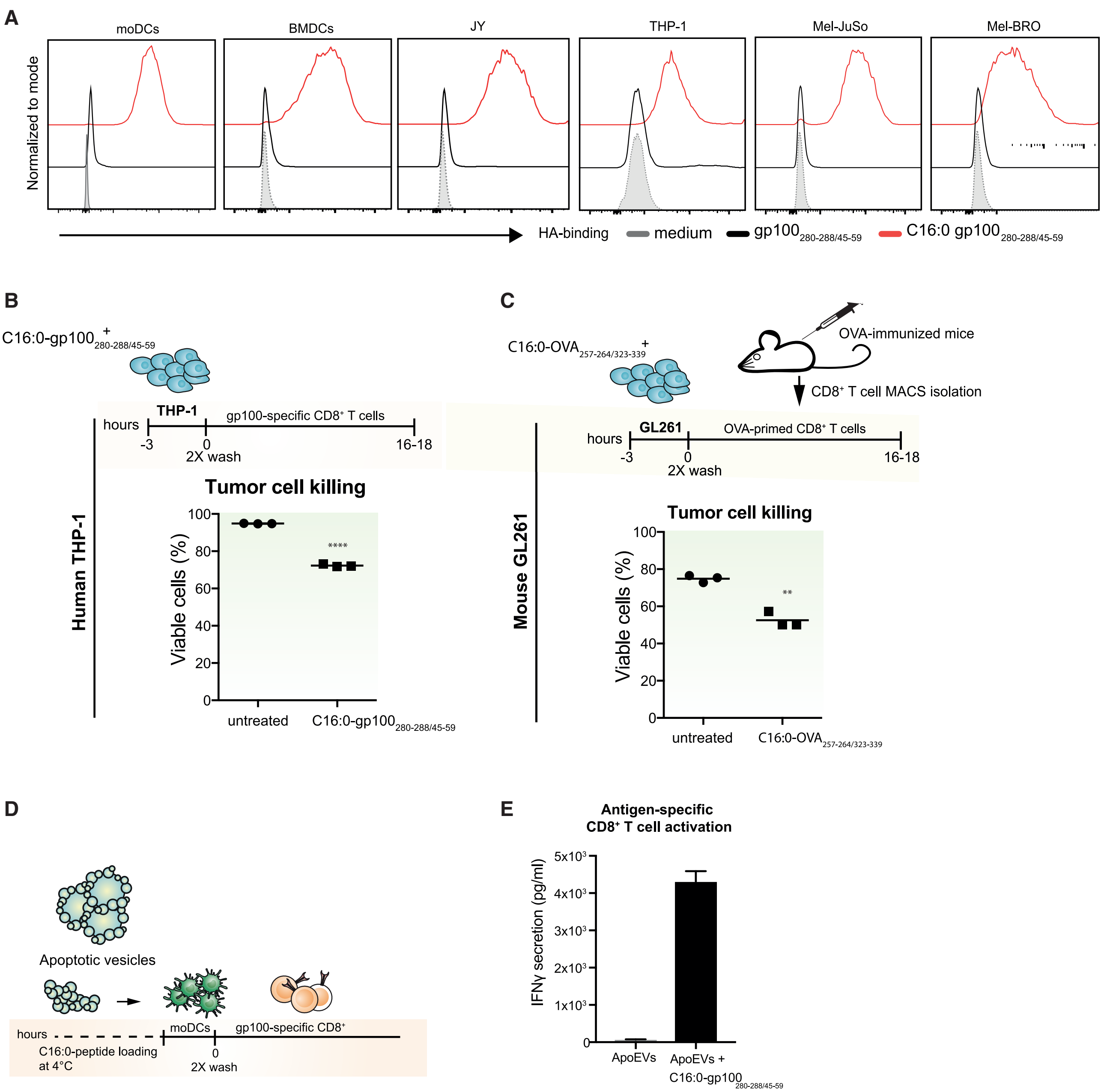

Figure 5. Exploiting the membrane-integrating capability of C16:0 peptides as a tool for antigen enrichment in tumor cells and tumor-derived apoptotic vesicles

(A) HA signal in DCs (humans and mice) and different tumor cell lines (JY, THP-1, Mel-JuSo, and Mel-BRO cells) that were pulsed with 10 μM $gp100_{280–288/45–59}$ or 16:0 $gp100_{280–288/45–59}$ for 45 min at 4°C. Data shown are representative of multiple individual experiments. (B) Viability of human THP-1 cells (leukemia) and (C) murine GL261 (glioblastoma) tumor cells that were pulsed with 10 μM C16:0 $gp100_{280–288/45–59}$ or C16:0 $OVA_{257–264/323–339}$ and co-cultured with antigen-specific T cells (gp100 $CD8^+$ T cells/$CD8^+$ T cells from OVA-immunized mice). Data show representative experiment measured in triplicate. (D) Overview of experimental setup for antigen enrichment of apoptotic extracellular vesicles. (E) IFNγ secretion by gp100-specific $CD8^+$ T cells after co-culture with moDCs that were incubated with apoptotic cell-derived extracellular vesicles (ApoEVs) which were first loaded with 10 μM C16:0 $gp100_{280–288/45–59}$. Data are presented as mean ± SEM of three individual experiments, measured in triplicate.

binding of palmitic acid to TLR2.[38] Antigens conjugated to Pam3 (CysSerLys4) have been shown to activate APCs through TLR2 and to induce enhanced T cell responses *in vivo*.[39–41] However, there is no consensus in the literature regarding the maturation capacity of mono-palmitoylated peptides, as they bear little structural resemblance with bacterial-derived TLR2 ligands,[42] and the bond between

the peptide and lipid tail and the site of attachment (N-terminal or C-terminal) differs between vaccine formulations.[43] Herein we report, in line with findings of Andrieu et al.,[24] that mono-palmitoylation of peptides did not have DC maturation properties and therefore C16:0 peptides could be paired with any adjuvant of choice. This is important to note, as specific TLR agonists can affect cross-presentation,[16,44,45] which could potentially influence T cell priming and further enhance peptide-based vaccination strategies. Also, pairing of C16:0 peptides with adjuvants that recruit moDCs to the site of injection such as AddaVax could be an interesting strategy.[46]

Using different inhibitors, we demonstrate that the processing of C16:0 peptides follows a different route from that of unmodified peptides. Clearly, processing of C16:0 peptides relied on proteasomal degradation in the cytosol, endosomal acidification, and vacuolar processing via cathepsin S. In our hands, BFA did not affect the processing of the C16:0 gp100 peptides for presentation to $CD8^+$ T cells, suggesting that loading of C16:0 gp100 on MHC class I does not rely on Golgi transport. Other studies have reported that processing of mono-palmitoylated antigens was exclusively dependent on proteasomal degradation[19] and also dependent on Golgi transport,[24] which is in contrast with our findings. A possible explanation could be that in both studies a palmitic acid lysine building block was used compared to the direct N-terminal palmitic acid conjugation used in our study. This discrepancy highlights once again that the chemical formulation is critical for determining the intracellular fate of the palmitoylated peptide. In accordance, Verheul et al.[23] showed that the spacer sequence between the epitope sequence and palmitic acid is causal for its immunogenicity. The question therefore remains what exact formulation and which corresponding processing route will ultimately lead to the most efficient epitope presentation into MHC class I and thus poses the best strategy for peptide-based vaccines.

We show that C16:0-modified peptides efficiently bind the cell surface of cells and quickly enter the cell. Their internalization was not (recycling) receptor-mediated, as the uptake was a continuous process and the membrane binding was not inhibited after lowering expression of recycling receptors on the membrane by glucose depletion. Our data suggest that C16:0-modified peptides can incorporate into lipid bilayers, a mechanism that is also physiologically used by cells to shuttle proteins between lipid bilayers.[47] This notion was supported by the observation that C16:0 peptides were concurrently detected in the extracellular and the intracellular space, when kept at a low temperature.

The fact that C16:0-modified peptides target lipid bilayers instead of DC subset-restricted receptors potentially exploits the cross-priming capacities of multiple DC subsets, as C16:0-modified peptides accumulated in all skin-resident DCs. Interestingly, incubation of tumor cells with mono-palmitoylated peptides resulted in efficient recognition by CTLs followed by enhanced killing. Cross-presentation of exogenous peptides is normally reserved to DCs, but this finding suggests that cross-presentation of C16:0 peptides is independent of any DC-related function. As such, any cell type could be made to cross-present after loading with C16:0 peptides. To further utilize this feature, intra-tumoral injections of C16:0 peptides suggest an interesting mode of application, and future studies need to be conducted to fully prove the feasibility of this strategy. However, intra-tumoral injection of the H-2D^b-restricted HPV16 E749-57 peptide combined with the TLR3 ligand polyinosinic-polycytidylic acid (poly(I:C)) has already been shown to be effective. After intra-tumoral injection, increased frequencies of E7-specific tumor-infiltrating lymphocyte (TIL) $CD8^+$ T cells were detected and survival *in vivo* was prolonged compared to intra-muscular vaccination.[48] Additionally, a significant increase of E7 tetramer-negative $CD8^+$ TILs with a phenotype suggestive of antigen specificity (PD-1^+/Tim-3^+/Lag-3^+ or PD-1^+/Tim-3^+ or PD-1^+/Lag-3^+) was observed. The authors proposed that these activated E7 tetramer-negative $CD8^+$ TILs could be specific for other tumor-associated antigens (TAAs) or neo-antigens induced by epitope spreading. We hypothesize that this could have been accelerated by the enhanced E7/MHC class I complexes on the tumor cells, facilitating tumor killing and epitope spreading. Of course, side effects of intra-tumoral lipopeptide vaccination must be considered, since the membrane-integrating capacity of C16:0 peptides not only applies to DCs, but to all cells, which could lead to CTL responses against healthy cells, which should be avoided at all cost. However, in the study mentioned above no unwanted side effects of intra-tumoral HPV16 E749-57 peptide vaccination were reported. Importantly, the HPV16 peptide used in this vaccine consisted of only the MHC class I epitope (9-mer) and can therefore load any MHC class I expressing cell, without the need of classical cross-presentation, similar to C16:0 peptides. Intra-tumoral injection of C16:0 peptides might even be favorable over short peptide vaccination since presentation of short peptides in the vaccination site may result in defective local CTL responses.[49] Overall, this study paves the way for future studies with palmitoylated peptides to further look into the effect on the tumor microenvironment, CTL induction, and recognition of tumor cells by CTLs as a result of increased MHC class I/epitope complexes and CTL-mediated killing.

The addition of a single palmitic acid is a relatively easy and cost-effective modification. In theory, all peptides could be generated as a C16:0 peptide, including neo-antigen-derived epitopes. Tumors with a high mutational load are associated with increased T cell responses and improved clinical outcome,[50,51] as these T cells are not submissive to central tolerance. Neo-antigen vaccines induced relatively high response rates in melanoma patients[52,53] and are therefore gaining interest for therapeutic vaccination. Adding a single palmitic acid to neo-antigenic peptides could potentially further enhance these responses. In addition, tumor-derived vesicles are an excellent vehicle for antigen enrichment by mono-palmitoylated peptides, especially since they harbor a variety of tumor-specific (neo-)antigens, thereby potentially broadening the induced anti-tumor immunity and avoiding immune escape.[54] As such, we showed that ApoEVs can be easily loaded with C16:0 peptides, which resulted in enhanced antigen-specific T cell responses.

In summary, in this study we show that the modification of synthetic long peptides with a single palmitic acid results in efficient antigen

cross-presentation by APCs and non-professional APCs alike and induces potent *in vivo* tumor suppression. Moreover, the superior binding capacity of the C16:0 peptides does not only enhance antigen uptake by cells, but it also allows for antigen enrichment of vesicles or tumor cells and could therefore be used for the development of new therapeutic vaccination strategies. Thus, the addition of a single palmitic acid is a simple and relatively cheap antigen modification, resulting in a multi-purposed membrane-targeting moiety that can be used as a vaccine modality for the induction of T cell-mediated immunity and for antigen enrichment, leading to enhanced recognition of tumor target T cells by CTLs.

MATERIALS AND METHODS

Peptide synthesis and characterization

gp100$_{280–288/45–59}$ (VTHTYLEPGPVTANRQLYPEWTEAQRLDC) and OVA$_{257–264/323–339}$ (CEEKSIINFEKLISQAVHAAHAEINEAGR KEEK) peptides were produced by solid-phase peptide synthesis using 9-fluorenylmethoxycarbonyl (Fmoc)-based chemistry on a Symphony peptide synthesizer (Protein Technologies). Mono-palmitoylated peptides (C16:0) were prepared by the addition of palmitic anhydride (3 equiv, Sigma-Aldrich, Darmstadt, Germany) in dichloromethane (DCM) (Biosolve) to the last N-terminal amino acid of the peptide sequence using 4-(dimethylamino)pyridine

(DMAP) (Sigma-Aldrich, Darmstadt, Germany) as a catalyst. Next, cleavage of unmodified gp100 and OVA peptide and C16:0 gp100 and C16:0 OVA lipopeptide was performed with reagent K (trifluoroacetic acid [TFA] from Biosolve, Valkenswaard, the Netherlands; scavengers from Sigma-Aldrich, Darmstadt, Germany) and products were recovered after precipitation in diethyl ether (Iris Biotech, Marktredwitz, Germany). Purification of peptides and C16:0 peptides was performed on an Ultimate 3000 high-pressure liquid chromatography (HPLC) system (Thermo Fisher Scientific, Waltham, MA, USA) using a 22 × 250-mm Vydac MS214 prep C18 column (Grace Alltech, Worms, Germany, elution water/acetonitrile) or a 22 × 250-mm Vydac 214MS1022 prep C4 column (Grace Alltech, Worms, Germany, elution water/2-propanol), respectively. Purity of peptides and C16:0 peptides was confirmed by HPLC (Vydac 218MS C18 5-μm 4.6 × 250-mm column or ProSphere C4 330A 5-μm 4.6 × 150-mm column, respectively, both from Grace Alltech, Worms, Germany) and electrospray mass spectrometry (LCQ Deca XP ion trap mass spectrometer, Thermo Fisher Scientific, Waltham, MA, USA). For intracellular tracking the C16:0 gp100 peptide HA$_{98–106}$ sequence YPYDVPDYA was inserted before last cysteine on the C-terminus (VTHTYLEPGPVTANRQLYPEWTEAQRLD**YPYDVPDYA**C).

Cells and cell culture

Immature moDCs were generated from monocytes obtained from human peripheral blood mononuclear cells (PBMCs) isolated from buffy coats of healthy donors (Sanquin) by a sequential Lymphoprep (Thermo Fisher Scientific, Waltham, MA, USA) and Percoll (GE Healthcare, Chicago, IL, USA) gradient and cultured for 5–6 days in RPMI 1640 medium (Invitrogen) supplemented with 10% fetal calf serum (FCS) (Lonza, Basel, Switzerland), 100 U/mL penicillin/streptomycin (Lonza, Basel, Switzerland), and 2 mM glutamine (Lonza, Basel, Switzerland) (complete RPMI 1640) in the presence of recombinant human IL-4 (500 U/mL) and GM-CSF (800 U/mL) (ImmunoTools, Friesoythe, Germany). The THP-1 and JY cell lines were cultured in complete RPMI 1640, and the Mel-Juso and Mel-BRO cell lines were cultured in complete Iscove's modified Dulbecco's medium (IMDM). The gp100-specific HLA-DRB*0401-restricted T cell line Bridge gp:44 B8[55] and the retroviral T cell receptor (TCR)αβ-transduced T cell clone specific for the gp100$_{280–288}$ HLA-A2 minimal epitope[56] were cultured in Yssel's medium[57] supplemented with 1% human serum, penicillin, streptomycin, and glutamine.

Mice

Mice transgenic for OT-I TCR on the C57BL/6 background have been described previously.[58,59] *Batf3*-deficient mice were obtained from The Jackson Laboratory and have been described previously.[5] Transgenic and WT C57BL/6 mice were bred at the animal facility of VU University (Amsterdam, the Netherlands) under specific pathogen-free conditions and used at 8–16 weeks of age. All experiments were approved by the Animal Welfare Body (IvD) of the VU University and performed in accordance with national and international guidelines and regulations under the project license assessed by the Dutch Central Authority for Scientific Procedures on animals (CCD).

Confocal microscopy

To image cells pulsed with C16:0 peptides (C16:0 gp100 or C16:0 OVA), we used C16:0 peptides containing the HA amino acid sequence. AF647-labeled anti-HA (clone 6E2, Cell Signaling Technology, Danvers, MA, USA) Ab was used to visualize the location of the C16:0 peptides. Stained cells were fixed with 2% paraformaldehyde (PFA)/PBS and mounted on glass slides using a Shandon Cytospin (Marshall Scientific, Hampton, NH, USA). Subsequent imaging was performed using a Leica TCS SP8 with pulsed white-light laser (set at 50% power) and STED 3X module. DAPI was excited using the 405-nm UV laser (set at 2% power). Individual excitation of fluorochromes was adjusted and tailored to the label, with AF488 excited at 495 nm (20% power) and AF647 excited at 650 nm (25% power). For STED super-resolution using the HC PL APO CS2 ×100/1.40 oil immersion objective, AF647 fluorochrome was depleted using the 775-nm STED depletion laser set at 100% power. Acquisition of the AF647 signal was performed by the HyD detector set at 663–740 nm. For STED imaging a single-pixel resolution of 19 × 19 nm was used.

Binding and uptake experiments

Immature moDCs were incubated with 10 μM gp100 or C16:0 gp100 (both containing a HA tag) at 4°C. Subsequently, moDCs were transferred to 37°C, and on t = 15-, 30-, and 60-min samples were taken. Next, the cells were fixed on ice with 1%–4% PFA (Electron Microscopy Sciences, Hatfield, PA, USA) and after extensive washing stained for the detection of the HA tag with an AF647-anti-HA Ab (Cell Signaling Technology, Danvers, MA, USA, clone 6E2). For intracellular detection of the HA tag, moDCs were permeabilized

with 0.1% saponin prior to staining with anti-HA AF488. To analyze the effect of glucose depletion on C16:0 gp100 binding, moDCs were pre-treated with glucose depletion medium (RPMI 1640 glucose-free medium, supplemented with 50 mM 2-deoxy-D-glucose [Sigma, Darmstadt, Germany] and 0.02% sodium azide) for 30 min prior to (at 37°C) and during the 45-min antigen pulse at 4°C. Thereafter, cells were fixed and stained to detect the MR Ab (clone 19.2, BD Biosciences, San Jose, CA, USA) or HA tag (clone 6E2, Cell Signaling Technology, Danvers, MA, USA) on the cell surface. Stained cells were measured on an X20 LSRFortessa SORP flow cytometer (BD Biosciences, San Jose, CA, USA) or a FACSCalibur flow cytometer (BD Biosciences, San Jose, CA, USA), and data were analyzed by FlowJo software.

DC maturation assay

Immature moDCs were seeded in a 96-well U-bottom plate (Greiner Bio-One, Kremsmünster, Austria) at a density of 50 × 10^3 cells per well and pulsed with the different peptides or 100 ng/mL lipopolysaccharide (LPS) (InvivoGen, Toulouse, France). After 3 h, DCs were washed and cultured for an additional 16–18 h overnight (o/n). Maturation marker expression on the moDCs was detected using the following Abs: fluorescein isothiocyanate (FITC)-labeled anti-CD80 (clone 2D10, BioLegend, San Diego, CA, USA), PE-Cy7-labeled anti-CD83 (clone HB15e, eBioscience, San Diego, CA, USA), PE-labeled anti-CD86 (clone 2331 [FUN-1], BD Biosciences, San Jose, CA, USA), and Brilliant Violet (BV)510-labeled anti-HLA-DR (clone G46-6, BD Biosciences, San Jose, CA, USA). Stained cells were measured on an X20 LSRFortessa SORP flow cytometer (BD Biosciences, San Jose, CA, USA), and data were analyzed by FlowJo software.

Human antigen presentation experiments

Immature moDCs were seeded in 96-well plates (Greiner Bio-One, Kremsmünster, Austria) at 20 × 10^3 cells/well and incubated with 10, 1, or 0.1 μM gp100 or C16:0 gp100 peptide. After 3 h, moDCs were washed and co-cultured o/n with gp100-specific CD8$^+$ T cells[46] or the gp100-specific HLA-DRB1*0401-restricted T cell line Bridge gp:44 B8,[45] at a concentration of 10 × 10^4 cells per well (effector to target cell [E:T] ratio of 1:5). IFNγ secretion in the supernatant was measured by sandwich ELISA. To determine the effect of proteasomal and endosomal inhibitors, moDCs (20 × 10^3 cells/well) were incubated with chloroquine (50 μM, Sigma, Darmstadt, Germany), MG132 (50 μM, Selleckchem, Houston, TX, USA), bortezomib (10 nM, Sigma, Darmstadt, Germany), primaquine (50 μM, Sigma, Darmstadt, Germany), cathepsin S inhibitor (50 μM, Calbiochem, San Diego, CA, USA), leupeptin (15 μM, Calbiochem, San Diego, CA, USA), or vehicle (control is treated with the highest DMSO concentration used in the experiment; vehicle control peptide and C16:0 peptide are the same for all the inhibitors) for 30 min at 37°C prior and during the 3-h pulse with gp100, C16:0 gp100, or short peptide. After 3 h the DCs were washed and co-cultured with gp100-specific CD8$^+$ T cells[46] (10 × 10^4 cells per well, E:T ratio of 1:5). IFNγ secretion, as a measure of T cell activation, was analyzed by sandwich ELISA (eBioscience, San Diego, CA, USA).

Murine antigen presentation experiments

BMDCs were cultured as described by Lutz et al.[60] OT-I transgenic mice were sacrificed and spleens were mechanistically dissociated by mashing them through a 100-μm cell strainer. Red blood cells were lysed using ACK lysis buffer (0.15 M NH_4Cl, 10 mM $KHCO_3$, 0.1 mM EDTA) and washed before purification using MagniSort mouse CD8-negative isolation kits according to the manufacturer's instructions (eBioscience/Thermo Fisher Scientific, Waltham, MA, USA). Purified CD8$^+$ T cells were labeled using 2 μM CFSE (5-(and 6)-carboxyfluorescein diacetate, succinimidyl ester) and counted before co-culture. BMDCs and purified T cells were co-cultured for 3 days at 37°C, stained and measured on an X20 LSRFortessa SORP flow cytometer. Results were calculated and presented as percentage responding cells (calculated as (cells at the start of culture/number of cells that went into division) × 100).

Tumor-loading experiments

20 × 10^3 cells/well of human THP-1 cells and 20 × 10^3 cells/well of mouse GL261 cells were pulsed with peptide or C16:0 peptide for 3 h, then washed and co-cultured with human gp100-specific CD8$^+$ T cells or OVA-primed CD8$^+$ T cells from OVA-immunized mice or (E:T ratio of 1:5 and 1:2, respectively) o/n. The next day, the tumor cells were harvested (using EDTA) and stained with fixable viability dye (FVD) eFluor 780 (Thermo Fisher Scientific, Waltham, MA, USA), the amount of tumor kill was measured on an X20 LSRFortessa SORP flow cytometer (BD Biosciences, San Jose, CA, USA), and data were analyzed by FlowJo software.

Human skin explant experiments

Human skin explants from healthy donors were obtained within 24 h after surgery (Bergman Clinics, Bilthoven, the Netherlands). gp100 or C16:0 gp100 peptides were diluted in serum-free medium (for two donors the medium was supplemented with GM-CSF [262.5 U/mL], recombinant human [rh]IL-4 [112.5 U/mL], and injected intradermally [i.d.] [20 μL]). A punch biopsy (8 mm) was taken at the site of injection and cultured for 2 days in a 48-well plate containing 1 mL of complete IMDM supplemented with 10 μg/mL gentamycin. After 2 days the migrated cells were collected and stained with BV510-labeled anti-HLA-DR, AF700-labeled anti-CD14 (clone M5E2, Sony), allophycocyanin (APC)-labeled anti-CD1a (clone HI149; BD Biosciences, San Jose, CA, USA), BV421-labeled anti-EpCAM (clone EBA-1; BioLegend), AF488-labeled anti-HA and FVD (Thermo Fisher Scientific, Waltham, MA, USA), or co-cultured with gp100-specific CD8$^+$ T cells[56] (E:T ratio of 1:5) o/n. The stained cells were measured on an X20 LSRFortessa SORP flow cytometer (BD Biosciences, San Jose, CA, USA) and data were analyzed by FlowJo software. Informed consent from patients that underwent corrective breast or abdominal plastic surgery was obtained according to hospital guidelines and in accordance with the "Code for Proper Use of Human Tissues" as formulated by the Dutch Federation of Medical Scientific Organizations.

Isolation murine CD11c$^+$ splenic DCs from *Batf3* KO mice

Splenic CD11c$^+$ DCs from *Batf3* KO mice were isolated using a combination of enzymatic digestion and magnetic-activated cell sorting

(MACS) using a CD11c magnetic isolation kit according to the manufacturer's instructions (MagniSort, eBioscience/Thermo Fisher Scientific, Waltham, MA, USA). In short, mice were sacrificed and the spleens were isolated and cut into small pieces using sterile scissors in 385 μg/mL Liberase TL (2 Wünsch Units) and incubated for 20 min at 37°C. Enzymes were deactivated using ice-cold RPMI 1640 complete medium (10% FCS, 50 U/mL penicillin, 50 μg/mL streptomycin, HEPES/EDTA). After digestion, cells were run through a 100-μm cell strainer and extensively washed before MACS. Purity of $CD11c^+$ cells was typically >96% of alive cells.

Vaccination experiments

Mice were vaccinated s.c. with OVA or C16:0 OVA peptide supplemented with 25 μg of agonistic CD40 Ab (in-house, clone 1C10) in a maximum volume of 100 μL. Seven days after vaccination, mice were sacrificed and the spleens were obtained for further processing. The spleens were mashed through a 100 μm cell strainer and resuspended in ACK red blood cell lysis buffer. Lysis buffer was deactivated by ice-cold RPMI 1640 complete medium (10% FCS, 1% 50 U/mL penicillin, 50 μg/mL streptomycin). Next, splenocytes were counted and 3×10^6 cells were stained for 30 min at 4°C using directly labeled primary Abs and in the presence of 1 μg/mL anti-CD16/32 Ab to block Fc receptor binding. Antigen-specific $CD8^+$ T cells were detected by incubation with H-$2K^b$:SIINFEKL tetramers and CD8a Ab prior to staining with other Abs. After extensive washing with PBS, labeled cells were fixed with 1% PFA at 4°C for 15 min, washed, and measured on an X20 LSRFortessa SORP flow cytometer (BD Biosciences, San Jose, CA, USA) and data were analyzed by FlowJo software.

Tumor challenge and therapeutic vaccination

C57BL/6 animals were inoculated s.c. in the flank with 100 μL of PBS containing 3×10^5 B16-OVA cells (kind gift from Prof. T.N. Schumacher, Netherlands Cancer Institute) under anesthetic conditions using 2%–3% isoflurane. Tumor growth was monitored three times/week by digital caliper and calculated by: $\frac{4}{3} * \pi *$ abc (where a represents width of tumor/2, b represents length of tumor/2, and c represents average of a and b/2). Nine days after tumor inoculation all tumors were palpable (average size 20 mm^3) and mice were randomized into treatment groups. Mice were s.c. immunized with 30 nmol C16:0 or unmodified peptide supplemented with 25 μg of anti-CD40. An additional boost vaccination was given 14 days after the initial immunization. Mice were sacrificed when tumor reached a size >800 mm^3 or at the end of the study (day 49).

ApoEV induction and isolation protocol

Apoptosis was induced in Mel-JuSo cells by adding 20 nM proteasome inhibitor bortezomib (Sigma, Darmstadt, Germany) to the culture medium for 72 h. The ApoEVs were isolated after the induction apoptosis via differential centrifugation steps: 10 min at 400 × *g*, 20 min at 1,200 × *g*, and 30 min at 10,000 × **g** in order to remove cell debris (400 × *g* fraction) and larger ApoEVs (1,200 × *g* fraction).[32] All centrifugation steps were performed at 4°C. The pellet containing ApoEVs (10.000 × *g* fraction) was used for further experiments. Protein concentration was determined by a Bradford protein assay kit (Thermo Fisher Scientific, Waltham, MA, USA) according to the manufacturer's instructions (Thermo Fisher Scientific, Waltham, MA, USA).

Statistical analysis

Statistics were performed using GraphPad Prism 7 software. For the comparison of two groups a (paired) Student's t test was used. For more than two groups, a one-way ANOVA or two-way ANOVA was performed. Tumor growth was analyzed with a linear regression model. $^*p < 0.05$, $^{**}p < 0.01$, $^{***}p < 0.001$. Data are represented as mean ± SEM.

SUPPLEMENTAL INFORMATION

Supplemental information can be found online at https://doi.org/10.1016/j.omto.2021.04.009.

ACKNOWLEDGMENTS

We thank all members of the O|2 Flow Cytometry, AO2M Microscopy, GlycO2 peptide, and MO2Ab and Amsterdam Animal Research center (AARC) facilities of Amsterdam UMC, location VUmc, for technical support, peptide synthesis, and support with animal experiments. In addition, we thank Bergman clinics (Bilthoven, the Netherlands) for providing healthy donor skin. Special thanks to Deimante Normataite for providing maturation data on moDCs during her Master's apprenticeship, and to Hans J. van der Vliet for carefully reading and editing of the manuscript. This work was supported by Glycotreat ERC Advanced 339977 (to S.K.H., S.T.T.S., S.D., and Y.v.K.) and KWF VU2014-7200 (to D.A.S., T.D.d.G., and Y.v.K.).

AUTHOR CONTRIBUTIONS

S.K.H., S.T.T.S., D.A.S., J.J.G.-V., S.J.v.V., and Y.v.K. were responsible for design of the experiments; data collection was performed by S.K.H., D.A.S., S.T.T.S., S.D., L.J.W.K., R.v.d.V., and E.K.; peptides were synthesized by M.A. and H.K.; all authors participated in the data analysis and interpretation; S.K.H., D.A.S., S.T.T.S., S.J.v.V., T.D.d.G., and Y.v.K. wrote the article. Funding acquisition was executed by Y.v.K. and T.D.d.G. All authors have read and agreed to the published version of the manuscript.

DECLARATION OF INTERESTS

The authors declare no competing interests.

REFERENCES

1. Hodi, F.S., O'Day, S.J., McDermott, D.F., Weber, R.W., Sosman, J.A., Haanen, J.B., Gonzalez, R., Robert, C., Schadendorf, D., Hassel, J.C., et al. (2010). Improved survival with ipilimumab in patients with metastatic melanoma. N. Engl. J. Med. *363*, 711–723.

2. Wolchok, J.D., Kluger, H., Callahan, M.K., Postow, M.A., Rizvi, N.A., Lesokhin, A.M., Segal, N.H., Ariyan, C.E., Gordon, R.-A., Reed, K., et al. (2013). Nivolumab plus ipilimumab in advanced melanoma. N. Engl. J. Med. *369*, 122–133.

3. Larkin, J., Chiarion-Sileni, V., Gonzalez, R., Grob, J.J., Cowey, C.L., Lao, C.D., Schadendorf, D., Dummer, R., Smylie, M., Rutkowski, P., et al. (2015). Combined nivolumab and ipilimumab or monotherapy in untreated melanoma. N. Engl. J. Med. *373*, 23–34.

4. Steinman, R.M., and Banchereau, J. (2007). Taking dendritic cells into medicine. Nature *449*, 419–426.

5. Hildner, K., Edelson, B.T., Purtha, W.E., Diamond, M., Matsushita, H., Kohyama, M., Calderon, B., Schraml, B.U., Unanue, E.R., Diamond, M.S., et al. (2008). *Batf3* deficiency reveals a critical role for CD8α+ dendritic cells in cytotoxic T cell immunity. Science *322*, 1097–1100.

6. Segura, E., Durand, M., and Amigorena, S. (2013). Similar antigen cross-presentation capacity and phagocytic functions in all freshly isolated human lymphoid organ-resident dendritic cells. J. Exp. Med. *210*, 1035–1047.

7. Nizzoli, G., Krietsch, J., Weick, A., Steinfelder, S., Facciotti, F., Gruarin, P., Bianco, A., Steckel, B., Moro, M., Crosti, M., et al. (2013). Human CD1c+ dendritic cells secrete high levels of IL-12 and potently prime cytotoxic T-cell responses. Blood *122*, 932–942.

8. Tel, J., Schreibelt, G., Sittig, S.P., Mathan, T.S.M., Buschow, S.I., Cruz, L.J., Lambeck, A.J.A., Figdor, C.G., and de Vries, I.J.M. (2013). Human plasmacytoid dendritic cells efficiently cross-present exogenous Ags to CD8+ T cells despite lower Ag uptake than myeloid dendritic cell subsets. Blood *121*, 459–467.

9. Fehres, C.M., Kalay, H., Bruijns, S.C.M., Musaafir, S.A., Ambrosini, M., van Bloois, L., van Vliet, S.J., Storm, G., Garcia-Vallejo, J.J., and van Kooyk, Y. (2015). Cross-presentation through langerin and DC-SIGN targeting requires different formulations of glycan-modified antigens. J. Control. Release *203*, 67–76.

10. Fehres, C.M., van Beelen, A.J., Bruijns, S.C.M., Ambrosini, M., Kalay, H., Bloois, L.V., Unger, W.W.J., Garcia-Vallejo, J.J., Storm, G., de Gruijl, T.D., and Kooyk, Y.V. (2015). In situ delivery of antigen to DC-SIGN+CD14+ dermal dendritic cells results in enhanced CD8+ T-cell responses. J. Invest. Dermatol. *135*, 2228–2236.

11. Tang-Huau, T.L., Gueguen, P., Goudot, C., Durand, M., Bohec, M., Baulande, S., Pasquier, B., Amigorena, S., and Segura, E. (2018). Human in vivo-generated monocyte-derived dendritic cells and macrophages cross-present antigens through a vacuolar pathway. Nat. Commun. *9*, 2570.

12. Bonifaz, L., Bonnyay, D., Mahnke, K., Rivera, M., Nussenzweig, M.C., and Steinman, R.M. (2002). Efficient targeting of protein antigen to the dendritic cell receptor DEC-205 in the steady state leads to antigen presentation on major histocompatibility complex class I products and peripheral CD8+ T cell tolerance. J. Exp. Med. *196*, 1627–1638.

13. Unger, W.W., Mayer, C.T., Engels, S., Hesse, C., Perdicchio, M., Puttur, F., Streng-Ouwehand, I., Litjens, M., Kalay, H., Berod, L., et al. (2015). Antigen targeting to dendritic cells combined with transient regulatory T cell inhibition results in long-term tumor regression. Oncoimmunology *4*, e970462.

14. Schreibelt, G., Klinkenberg, L.J.J., Cruz, L.J., Tacken, P.J., Tel, J., Kreutz, M., Adema, G.J., Brown, G.D., Figdor, C.G., and de Vries, I.J. (2012). The C-type lectin receptor CLEC9A mediates antigen uptake and (cross-)presentation by human blood BDCA3+ myeloid dendritic cells. Blood *119*, 2284–2292.

15. van Dinther, D., Stolk, D.A., van de Ven, R., van Kooyk, Y., de Gruijl, T.D., and den Haan, J.M.M. (2017). Targeting C-type lectin receptors: a high-carbohydrate diet for dendritic cells to improve cancer vaccines. J. Leukoc. Biol. *102*, 1017–1034.

16. Horrevorts, S.K., Duinkerken, S., Bloem, K., Secades, P., Kalay, H., Musters, R.J., van Vliet, S.J., García-Vallejo, J.J., and van Kooyk, Y. (2018). Toll-like receptor 4 triggering promotes cytosolic routing of DC-SIGN-targeted antigens for presentation on MHC class I. Front. Immunol. *9*, 1231.

17. Tacken, P.J., Ginter, W., Berod, L., Cruz, L.J., Joosten, B., Sparwasser, T., Figdor, C.G., and Cambi, A. (2011). Targeting DC-SIGN via its neck region leads to prolonged antigen residence in early endosomes, delayed lysosomal degradation, and cross-presentation. Blood *118*, 4111–4119.

18. Hopp, T.P. (1984). Immunogenicity of a synthetic HBsAg peptide: Enhancement by conjugation to a fatty acid carrier. Mol. Immunol. *21*, 13–16.

19. Song, Y.C., Chou, A.H., Homhuan, A., Huang, M.H., Chiang, S.K., Shen, K.Y., Chuang, P.W., Leng, C.H., Tao, M.H., Chong, P., and Liu, S.J. (2011). Presentation of lipopeptide by dendritic cells induces anti-tumor responses via an endocytosis-independent pathway in vivo. J. Leukoc. Biol. *90*, 323–332.

20. Xu, D.H., Zhou, C.H., Xia, Y.P., Qiu, Z.Y., Wu, Y.Z., Jia, Z.C., and Zhou, W. (2007). Cytotoxic T lymphocyte response induced by an improved synthetic lipopeptide vaccine against cervical cancer. Acta Pharmacol. Sin. *28*, 695–702.

21. Gras-Masse, H. (2001). Single-chain lipopeptide vaccines for the induction of virus-specific cytotoxic T cell responses in randomly selected populations. Mol. Immunol. *38*, 423–431.

22. Cai, H., Sun, Z.-Y., Chen, M.-S., Zhao, Y.-F., Kunz, H., and Li, Y.-M. (2014). Synthetic multivalent glycopeptide-lipopeptide antitumor vaccines: impact of the cluster effect on the killing of tumor cells. Angew. Chem. Int. Ed. Engl. *53*, 1699–1703.

23. Verheul, A.F., Udhayakumar, V., Jue, D.L., Wohlhueter, R.M., and Lal, A.A. (1995). Monopalmitic acid-peptide conjugates induce cytotoxic T cell responses against malarial epitopes: importance of spacer amino acids. J. Immunol. Methods *182*, 219–226.

24. Andrieu, M., Desoutter, J.-F., Loing, E., Gaston, J., Hanau, D., Guillet, J.-G., and Hosmalin, A. (2003). Two human immunodeficiency virus vaccinal lipopeptides follow different cross-presentation pathways in human dendritic cells. J. Virol. *77*, 1564–1570.

25. Babu, J.S., Nair, S., Kanda, P., and Rouse, B.T. (1995). Priming for virus-specific CD8+ but not CD4+ cytotoxic T lymphocytes with synthetic lipopeptide is influenced by acylation units and liposome encapsulation. Vaccine *13*, 1669–1676.

26. Nicholas, D.A., Zhang, K., Hung, C., Glasgow, S., Aruni, A.W., Unternaehrer, J., Payne, K.J., Langridge, W.H.R., and De Leon, M. (2017). Palmitic acid is a Toll-like receptor 4 ligand that induces human dendritic cell secretion of IL-1β. PLoS ONE *12*, e0176793.

27. de Gruijl, T.D., Sombroek, C.C., Lougheed, S.M., Oosterhoff, D., Buter, J., van den Eertwegh, A.J.M., Scheper, R.J., and Pinedo, H.M. (2006). A postmigrational switch among skin-derived dendritic cells to a macrophage-like phenotype is predetermined by the intracutaneous cytokine balance. J. Immunol. *176*, 7232–7242.

28. Duinkerken, S., Lübbers, J., Stolk, D., Vree, J., Ambrosini, M., Kalay, H., and van Kooyk, Y. (2019). Comparison of intradermal injection and epicutaneous laser microporation for antitumor vaccine delivery in a human skin explant model. bioRxiv. https://doi.org/10.1101/861930.

29. Embgenbroich, M., and Burgdorf, S. (2018). Current concepts of antigen cross-presentation. Front. Immunol. *9*, 1643.

30. Nuchtern, J.G., Bonifacino, J.S., Biddison, W.E., and Klausner, R.D. (1989). Brefeldin A implicates egress from endoplasmic reticulum in class I restricted antigen presentation. Nature *339*, 223–226.

31. van der Ende, A., du Maine, A., Schwartz, A.L., and Strous, G.J. (1989). Effect of ATP depletion and temperature on the transferrin-mediated uptake and release of iron by BeWo choriocarcinoma cells. Biochem. J. *259*, 685–692.

32. Horrevorts, S.K., Stolk, D.A., van de Ven, R., Hulst, M., van Het Hof, B., Duinkerken, S., Heineke, M.H., Ma, W., Dusoswa, S.A., Nieuwland, R., et al. (2019). Glycan-modified apoptotic melanoma-derived extracellular vesicles as antigen source for anti-tumor vaccination. Cancers (Basel) *11*, 1–18.

33. Steller, M.A., Gurski, K.J., Murakami, M., Daniel, R.W., Shah, K.V., Celis, E., Sette, A., Trimble, E.L., Park, R.C., and Marincola, F.M. (1998). Cell-mediated immunological responses in cervical and vaginal cancer patients immunized with a lipidated epitope of human papillomavirus type 16 E7. Clin. Cancer Res. *4*, 2103–2109.

34. Launay, O., Surenaud, M., Desaint, C., Ben Hamouda, N., Pialoux, G., Bonnet, B., Poizot-Martin, I., Gonzales, G., Cuzin, L., Bourgault-Villada, I., et al. (2013). Long-term CD4+ and CD8+ T-cell responses induced in HIV-uninfected volunteers following intradermal or intramuscular administration of an HIV-lipopeptide vaccine (ANRS VAC16). Vaccine *31*, 4406–4415.

35. Salmon-Céron, D., Durier, C., Desaint, C., Cuzin, L., Surenaud, M., Hamouda, N.B., Lelièvre, J.D., Bonnet, B., Pialoux, G., Poizot-Martin, I., et al.; ANRS VAC18 trial group (2010). Immunogenicity and safety of an HIV-1 lipopeptide vaccine in healthy adults: A phase 2 placebo-controlled ANRS trial. AIDS *24*, 2211–2223.

36. Le Gal, F.A., Prevost-Blondel, A., Lengagne, R., Bossus, M., Farace, F., Chaboissier, A., Gras-Masse, H., Engelhard, V.H., Guillet, J.G., and Gahéry-Ségard, H. (2002). Lipopeptide-based melanoma cancer vaccine induced a strong MART-27-35-cytotoxic T lymphocyte response in a preclinal study. Int. J. Cancer *98*, 221–227.

37. Gahéry-Ségard, H., Pialoux, G., Figueiredo, S., Igéa, C., Surenaud, M., Gaston, J., Gras-Masse, H., Lévy, J.P., and Guillet, J.G. (2003). Long-term specific immune responses induced in humans by a human immunodeficiency virus type 1 lipopeptide vaccine: characterization of CD8+-T-cell epitopes recognized. J. Virol. *77*, 11220–11231.

38. Zhu, X., Ramos, T.V., Gras-Masse, H., Kaplan, B.E., and BenMohamed, L. (2004). Lipopeptide epitopes extended by an N^{ε}-palmitoyl-lysine moiety increase uptake and maturation of dendritic cells through a Toll-like receptor-2 pathway and trigger a Th1-dependent protective immunity. Eur. J. Immunol. *34*, 3102–3114.

39. Hertz, C.J., Kiertscher, S.M., Godowski, P.J., Bouis, D.A., Norgard, M.V., Roth, M.D., and Modlin, R.L. (2001). Microbial lipopeptides stimulate dendritic cell maturation via Toll-like receptor 2. J. Immunol. *166*, 2444–2450.

40. Khan, S., Weterings, J.J., Britten, C.M., de Jong, A.R., Graafland, D., Melief, C.J.M., van der Burg, S.H., van der Marel, G., Overkleeft, H.S., Filippov, D.V., and Ossendorp, F. (2009). Chirality of TLR-2 ligand Pam3CysSK4 in fully synthetic peptide conjugates critically influences the induction of specific $CD8^+$ T-cells. Mol. Immunol. *46*, 1084–1091.

41. Zom, G.G., Khan, S., Britten, C.M., Sommandas, V., Camps, M.G.M., Loof, N.M., Budden, C.F., Meeuwenoord, N.J., Filippov, D.V., van der Marel, G.A., et al. (2014). Efficient induction of antitumor immunity by synthetic toll-like receptor ligand-peptide conjugates. Cancer Immunol. Res. *2*, 756–764.

42. Basto, A.P., and Leitão, A. (2014). Targeting TLR2 for vaccine development. J. Immunol. Res. *2014*, 619410.

43. Surenaud, M., Lacabaratz, C., Zurawski, G., and Lévy, Y. (2017). Development of an epitope-based HIV-1 vaccine strategy from HIV-1 lipopeptide to dendritic-based vaccines. Expert Rev. Vaccines *16*, 955–972.

44. Nair-Gupta, P., Baccarini, A., Tung, N., Seyffer, F., Florey, O., Huang, Y., Banerjee, M., Overholtzer, M., Roche, P.A., Tampé, R., et al. (2014). TLR signals induce phagosomal MHC-I delivery from the endosomal recycling compartment to allow cross-presentation. Cell *158*, 506–521.

45. Gil-torregrosa, B.C., Lennon-dum, A.M., and Kessler, B. (2004). Control of cross-presentation during dendritic cell maturation. Eur. J. Immunol. *34*, 398–407.

46. Schetters, S.T.T., Kruijssen, L.J.W., Crommentuijn, M.H.W., Kalay, H., den Haan, J.M.M., and van Kooyk, Y. (2020). Immunological dynamics after subcutaneous immunization with a squalene-based oil-in-water adjuvant. FASEB J. *34*, 12406–12418.

47. Aicart-Ramos, C., Valero, R.A., and Rodriguez-Crespo, I. (2011). Protein palmitoylation and subcellular trafficking. Biochim. Biophys. Acta *1808*, 2981–2994.

48. Ishida, E., Lee, J., Campbell, J.S., Chakravarty, P.D., Katori, Y., Ogawa, T., Johnson, L., Mukhopadhyay, A., Faquin, W.C., Lin, D.T., et al. (2019). Intratumoral delivery of an HPV vaccine elicits a broad anti-tumor immune response that translates into a potent anti-tumor effect in a preclinical murine HPV model. Cancer Immunol. Immunother. *68*, 1273–1286.

49. Hailemichael, Y., Dai, Z., Jaffarzad, N., Ye, Y., Medina, M.A., Huang, X.-F., Dorta-Estremera, S.M., Greeley, N.R., Nitti, G., Peng, W., et al. (2013). Persistent antigen at vaccination sites induces tumor-specific $CD8^+$ T cell sequestration, dysfunction and deletion. Nat. Med. *19*, 465–472.

50. Rooney, M.S., Shukla, S.A., Wu, C.J., Getz, G., and Hacohen, N. (2015). Molecular and genetic properties of tumors associated with local immune cytolytic activity. Cell *160*, 48–61.

51. Giannakis, M., Mu, X.J., Shukla, S.A., Qian, Z.R., Cohen, O., Nishihara, R., Bahl, S., Cao, Y., Amin-Mansour, A., Yamauchi, M., et al. (2016). Genomic correlates of immune-cell infiltrates in colorectal carcinoma. Cell Rep. *15*, 857–865.

52. Sahin, U., Derhovanessian, E., Miller, M., Kloke, B.P., Simon, P., Löwer, M., Bukur, V., Tadmor, A.D., Luxemburger, U., Schrörs, B., et al. (2017). Personalized RNA mutanome vaccines mobilize poly-specific therapeutic immunity against cancer. Nature *547*, 222–226.

53. Ott, P.A., Hu, Z., Keskin, D.B., Shukla, S.A., Sun, J., Bozym, D.J., Zhang, W., Luoma, A., Giobbie-Hurder, A., Peter, L., et al. (2017). An immunogenic personal neoantigen vaccine for patients with melanoma. Nature *547*, 217–221.

54. Ruben, J.M., van den Ancker, W., Bontkes, H.J., Westers, T.M., Hooijberg, E., Ossenkoppele, G.J., de Gruijl, T.D., and van de Loosdrecht, A.A. (2014). Apoptotic blebs from leukemic cells as a preferred source of tumor-associated antigen for dendritic cell-based vaccines. Cancer Immunol. Immunother. *63*, 335–345.

55. Aarnoudse, C.A., Bax, M., Sánchez-Hernández, M., García-Vallejo, J.J., and van Kooyk, Y. (2008). Glycan modification of the tumor antigen gp100 targets DC-SIGN to enhance dendritic cell induced antigen presentation to T cells. Int. J. Cancer *122*, 839–846.

56. Schaft, N., Willemsen, R.A., de Vries, J., Lankiewicz, B., Essers, B.W.L., Gratama, J.W., Figdor, C.G., Bolhuis, R.L.H., Debets, R., and Adema, G.J. (2003). Peptide fine specificity of anti-glycoprotein 100 CTL is preserved following transfer of engineered TCRαβ genes into primary human T lymphocytes. J. Immunol. *170*, 2186–2194.

57. Yssel, H., De Vries, J.E., Koken, M., Van Blitterswijk, W., and Spits, H. (1984). Serum-free medium for generation and propagation of functional human cytotoxic and helper T cell clones. J. Immunol. Methods *72*, 219–227.

58. Hogquist, K.A., Jameson, S.C., Heath, W.R., Howard, J.L., Bevan, M.J., and Carbone, F.R. (1994). T cell receptor antagonist peptides induce positive selection. Cell *76*, 17–27.

59. Barnden, M.J., Allison, J., Heath, W.R., and Carbone, F.R. (1998). Defective TCR expression in transgenic mice constructed using cDNA-based α- and β-chain genes under the control of heterologous regulatory elements. Immunol. Cell Biol. *76*, 34–40.

60. Lutz, M.B., Kukutsch, N., Ogilvie, A.L.J., Rössner, S., Koch, F., Romani, N., and Schuler, G. (1999). An advanced culture method for generating large quantities of highly pure dendritic cells from mouse bone marrow. J. Immunol. Methods *223*, 77–92.

OMTO, Volume *21*

Supplemental information

Palmitoylated antigens for the induction of anti-tumor CD8$^+$ T cells and enhanced tumor recognition

Dorian A. Stolk, Sophie K. Horrevorts, Sjoerd T.T. Schetters, Laura J.W. Kruijssen, Sanne Duinkerken, Eelco Keuning, Martino Ambrosini, Hakan Kalay, Rieneke van de Ven, Juan J. Garcia-Vallejo, Tanja D. de Gruijl, Sandra J. van Vliet, and Yvette van Kooyk

Supplementary Figure 1

A

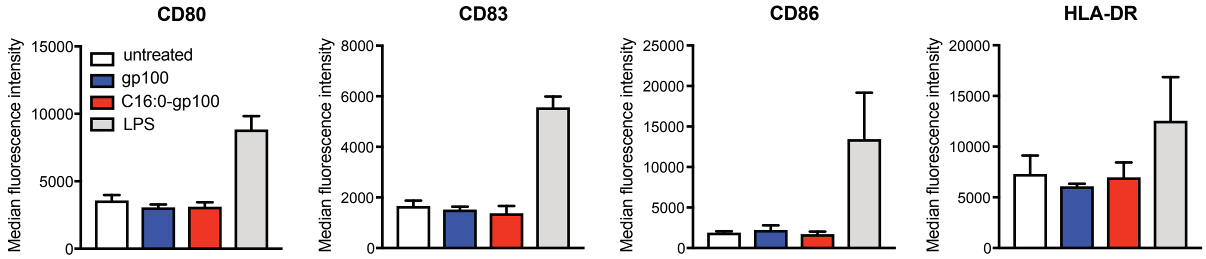

Figure S1. C16:0 peptide does not induce DC maturation (A) human moDC were incubated with unmodified peptide, C16:0 peptide, medium or LPS for 3hrs. Cells were washed and cultured o/n and stained for different maturation markers.

Supplementary Figure 2

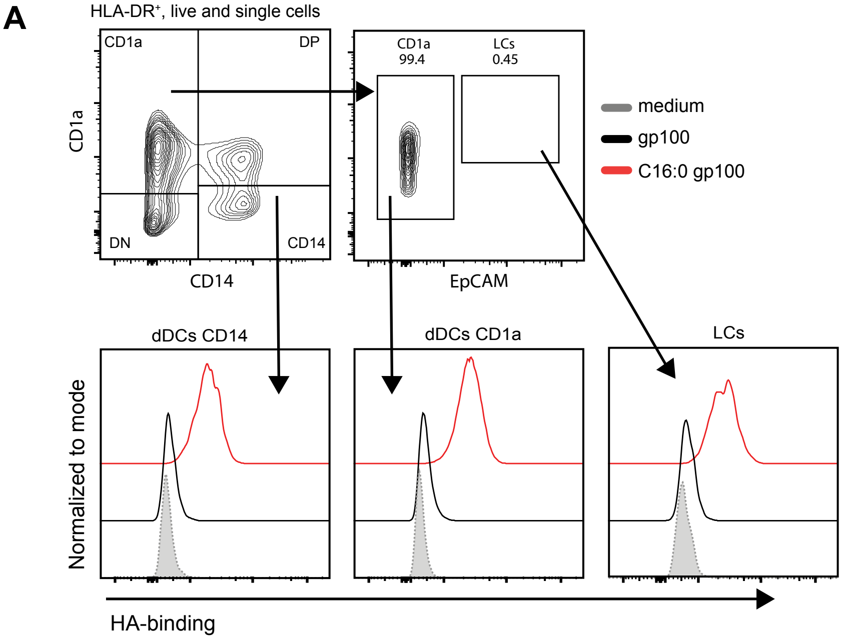

Figure S2. HA-tag detection in human skin crawl out dDCs. (A) Ex vivo human skin was intra-dermally injected with 30 μM unmodified gp100 or C16:0- p100 peptide in serum free medium. Punch biopsies were taken at the site of injection and cultured for 2 days. The crawl out cells were collected and stained for HLA-DR, CD1a, CD14, EpCAM and the HA-tag. Figure is representative for multiple individual experiments.

Supplementary Figure 3

A

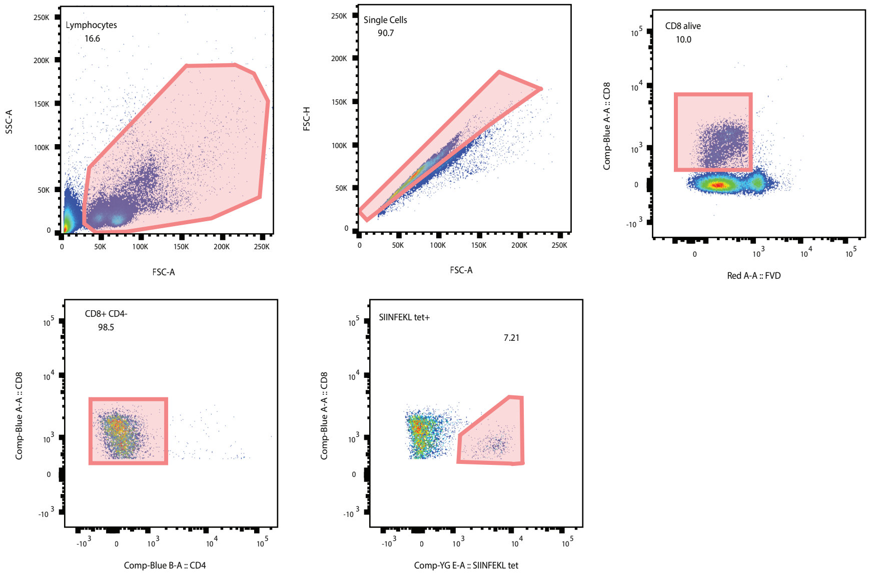

Figure S3. Gating strategy SIINFEKL+ CD8+ T-cells (A) Mouse blood or spleen was processed and stained with an antibody mxi containing CD8, CD4, Fixable Viability Dye (FVD), SIINFEKL tetramer.
